# Dietary ascorbic acid and subsequent change in body weight and waist circumference: associations may depend on genetic predisposition to obesity - a prospective study of three independent cohorts

**DOI:** 10.1186/1475-2891-13-43

**Published:** 2014-05-03

**Authors:** Sofus C Larsen, Lars Ängquist, Tarunveer Singh Ahluwalia, Tea Skaaby, Nina Roswall, Anne Tjønneland, Jytte Halkjær, Kim Overvad, Oluf Pedersen, Torben Hansen, Allan Linneberg, Lise Lotte N Husemoen, Ulla Toft, Berit L Heitmann, Thorkild IA Sørensen

**Affiliations:** 1 Institute of Preventive Medicine, Bispebjerg and Frederiksberg Hospitals, the Capital Region, Nordre Fasanvej 57, Hovedvejen, entrance 5, ground floor, 2000, Frederiksberg Copenhagen, Denmark; 2 The Novo Nordisk Foundation Center for Basic Metabolic Research, Section on Metabolic Genetics, Faculty of Health and Medical Sciences, University of Copenhagen, Copenhagen, Denmark; 3 Copenhagen Prospective Studies on Asthma in Childhood, Health Sciences, University of Copenhagen & Danish Pediatric Asthma Center, Copenhagen University Hospital, Gentofte, Denmark; 4 Research Centre for Prevention and Health, Glostrup University Hospital, Glostrup, Denmark; 5 Danish Cancer Society Research Center, Copenhagen, Denmark; 6 Section of Epidemiology, Department of Public Health, Aarhus University, Aarhus, Denmark; 7 Department of Cardiology, Aalborg University Hospital, Aalborg, Denmark; 8 The National Institute of Public Health, University of Southern Denmark, Copenhagen, Denmark; 9 The Boden Institute of Obesity, Nutrition, Exercise & Eating Disorders, The University of Sydney, Sydney, Australia

**Keywords:** Ascorbic acid, Genetic predisposition, Gene-diet interaction, Weight change

## Abstract

**Background:**

Cross-sectional data suggests that a low level of plasma ascorbic acid positively associates with both Body Mass Index (BMI) and Waist Circumference (WC). This leads to questions about a possible relationship between dietary intake of ascorbic acid and subsequent changes in anthropometry, and whether such associations may depend on genetic predisposition to obesity. Hence, we examined whether dietary ascorbic acid, possibly in interaction with the genetic predisposition to a high BMI, WC or waist-hip ratio adjusted for BMI (WHR), associates with subsequent annual changes in weight (∆BW) and waist circumference (∆WC).

**Methods:**

A total of 7,569 participants’ from MONICA, the Diet Cancer and Health study and the INTER99 study were included in the study. We combined 50 obesity associated single nucleotide polymorphisms (SNPs) in four genetic scores: a score of all SNPs and a score for each of the traits (BMI, WC and WHR) with which the SNPs associate. Linear regression was used to examine the association between ascorbic acid intake and ΔBW or ΔWC. SNP-score × ascorbic acid interactions were examined by adding product terms to the models.

**Results:**

We found no significant associations between dietary ascorbic acid and ∆BW or ∆WC. Regarding SNP-score × ascorbic acid interactions, each additional risk allele of the 14 WHR associated SNPs associated with a ∆WC of 0.039 cm/year (P = 0.02, 95% CI: 0.005 to 0.073) per 100 mg/day higher ascorbic acid intake. However, the association to ∆WC only remained borderline significant after adjustment for ∆BW.

**Conclusion:**

In general, our study does not support an association between dietary ascorbic acid and ∆BW or ∆WC, but a diet with a high content of ascorbic acid may be weakly associated to higher WC gain among people who are genetically predisposed to a high WHR. However, given the quite limited association any public health relevance is questionable.

## Introduction

A higher occurrence of nutritional deficiencies among obese may seem contradictory in light of excess calorie intake. Nevertheless, micronutrient deficiencies have been found in obese individuals worldwide, and it has been suggested that some of these could have an impact on weight loss [1]. Thus, cross-sectional studies suggest that a low level of plasma ascorbic acid is associated with both higher body mass index (BMI) and waist circumference (WC) [2-4], but prospective data is lacking.

In addition to a cross-sectional relationship, however, a double blind placebo controlled trial among 38 obese females demonstrated that supplementation with 3 g of ascorbic acid per day improved weight loss compared to placebo (2.53 versus 0.95 kg, p = 0.015) during a 6-week period [5]. A possible explanation for this could be that ascorbic acid is a cofactor in the biosynthesis of carnitine, a metabolite necessary for the oxidation of fatty acids [6]. Consequently, a reduction in the ability to oxidize fat may be one mechanism behind the inverse relationship between ascorbic acid and obesity [7].

Thus, in spite of some indications of associations [2-5] and plausible biological mechanisms behind [6,7], the evidence of a causal relationship between intake of ascorbic acid and subsequent change in body weight (BW) or WC is weak. One reason for this could be interaction with genetic variants, causing ascorbic acid to mainly play a role in body weight regulation among individuals with a genetic predisposition to obesity. The growing number of obesity-associated SNPs identified by genome wide association studies (GWAS) [8-20] provides a unique opportunity to investigate this hypothesis.

Hence, the aim of our study was to examine whether dietary ascorbic acid, possibly in interaction with genetic predisposition to higher BMI, WC or waist-hip ratio adjusted for BMI (WHR), associates with annual change in body weight (ΔBW, kg/year) and waist circumference (∆WC, cm/year).

## Methods

### Study population

Our study is based on participants from the Danish part of the MONICA study [21], the Diet, Cancer and Health (DCH) study [22] and the INTER99 study [23], each of which are described in the following.

### MONICA

This cohort consists of a random subset of 4,581 men and women born in 1922, 1932 in 1942 and 1952, who were selected from residents of 11 surrounding municipalities in the former Copenhagen County. In 1982–83, a total of 3,608 (78.8%) of these participants took part in a health examination, including measurement of BW, height, dietary intake and blood sampling. Five years later, during 1987–88, another invitation was sent to all living participants and 2,987 men and women participated in both the first and second health examinations [24]. A total of 1,852 participants completed a seven-day food record in 1982–83 [25], 1,578 of these had complete information on covariates as well as repeated measures of BW and 1,426 of these had information on genetic variants.

For this study, we further excluded participants with prevalent cancer (n = 16), cardiovascular disease (n = 61) or self-reported diabetes (n = 20). The final study population consisted of 1,329 healthy participants with information on diet, genes, baseline and follow-up BW as well as information on potential confounders.

### The diet cancer and health (DCH) study

During 1993–97, a total of 160,725 Danish men and women living in Copenhagen and Aarhus, between 50 and 64 years of age, born in Denmark and with no diagnosis of cancer registered in the Danish Cancer registry were invited to participate in DCH. Of these, a total of 57,053 (35%) accepted the invitation. These participants completed a lifestyle questionnaire, and a 192 item semi-quantitative food frequency questionnaire (FFQ) to assess the average intake of food during the last year. Furthermore, a follow up study was conducted during 1999 to 2002 which included self-administered questionnaires on diet, lifestyle and self-measured anthropometry.

Data used in this particular study is based on two samples of 1,200 BW gainers and 1,209 randomly selected control cohort/sample individuals. These participants had no cancer, cardiovascular disease or diabetes at baseline and follow-up, stable smoking habits, an annual weight gain not more than 5 kg/year, age <60 years at baseline and <65 years at follow-up. BW gainers were defined as the individuals who experienced the greatest degree of annual weight gain during follow-up. They were identified in gender stratified analysis by using the residuals from a regression model of ∆BW on baseline values of age, BW and height, smoking status (current/former/never smokers), and follow-up time. From these models, a total of 600 male and 600 female BW gainers were selected. The random sample was based on the complete cohort. The overlap between BW gainers and random sample was small (n = 79), thus the size of the remaining (non-overlapping) random sample (n = 1,130) almost equaled the number of weight gainers (12). Of the 2,330 individuals, we had information on diet, genes, changes in anthropometry and selected covariates on 2,167 men and women (2,128 in the analysis of ∆WC). However, 278 of these only had information on FTO (rs9939609).

### INTER99

A population-based randomized controlled trial (CT00289237, ClinicalTrials.gov) initiated in 1999. This year, an age- and sex- stratified random sample of 13,016 men and women born in 1939–40, 1944–45, 1949–50, 1954–55, 1959–60, 1964–65, 1969–70, living in 11 municipalities in the former Copenhagen County was drawn from the Civil Registration System and invited to a health examination. Of the 13,016 participants, a total of 12,934 were eligible for invitation, and, of these, 6,784 (52.5%) participated. Dietary intake was assessed through FFQ [26]. Design and methods used in the study have been described in detail elsewhere [23,27]. In 2004, all participants from the baseline examination were re-invited for a follow-up study where the baseline examination programme was repeated [28]. Information on diet, genes, baseline and follow-up anthropometric measures as well as information on potential confounders was available on 4,574 subjects. For the present study, we excluded participants with prevalent cancer (n = 87), cardiovascular disease (n = 320) or self-reported diabetes (n = 94) and ended up with 4,073 participants (3,536 participants in the analysis of ∆WC).

All procedures in the three studies were in accordance with the Helsinki Declaration and all participants provided written informed consent.

### Assessment of dietary intake

As described in detail elsewhere, information about the participants' dietary intake was collected using a validated seven-day food record in MONICA [25] and the same validated FFQ in DCH [29,30] and INTER99 [26]. From this information, daily consumption of foods and nutrients were calculated for each participant using the software program *DANKOST*[31] in MONICA and *FoodCalc*[32] in DCH and INTER99. Both DANKOST and FoodCalc are based on the official Danish food composition tables (http://www.foodcomp.dk). Participants' daily intake of ascorbic acid was then calculated and included in the analysis as a continuous variable (unit; mg/day).

### Assessment of covariates

All participants reported information on smoking status (never smoked, ex-smoker or current smoker). Likewise, information was gathered about consumption of alcohol and included in the analysis as a continuous variable (MJ/day). Regarding physical activity, the MONICA participants were asked to classify themselves into one of four groups 1) Almost completely inactive: sedentary activities such as reading, watching television and going to the movies. 2) Some physical activity: at least 4 hours weekly including for example walking, cycling, construction work, bowling and table tennis. 3) Regular hard activity at least 3 hours weekly, including for example swimming, tennis and badminton etc. or heavy gardening. 4) Hard activity: elite sports such as swimming, soccer, badminton or long distance running several times a week. In DCH the questionnaire was used to obtain information on duration and types of physical activity. From this information the validated Cambridge Physical Activity Index was calculated by combining occupational physical activity with time spent on cycling and sport in summer and winter [33]. Participants were then divided into four physical activity categories (inactive, moderately inactive, moderately active, and active). In INTER99 information on physical activity was based on two questions on commuting physical activity and leisure time physical activity. From these two questions, overall physical activity was calculated by summing response on commuting physical activity (converted into minutes per week using five day working week) and a leisure time physical activity variable (converted into minutes per week) [28]. From this variable, overall physical activity was grouped into four categories <2 h/week, 2–3.9 h/week, 4–6.9 h/week and ≥7 h/week. Education was assessed with questions about years of regular schooling in all three cohorts and classified with respect to having a school education above or below the primary level. Finally, we included information on the participants’ age and gender, and the women reported whether they had entered menopause.

### Assessment of anthropometric measures

At baseline, height was measured to the nearest 0.5 cm and BW to the nearest 0.1 kg in all three cohorts. Likewise, WC was measured horizontally midway between the lower rib margin and the iliac crest to the nearest 1 cm in DCH and INTER99. We did not have measures of WC on enough participants to include this measure in the analysis of the MONICA participants.

At follow-up, the baseline procedure was repeated for MONICA and INTER99 participants. Regarding the follow-up measures in DCH, the participants received a self-administrated questionnaire and reported their weight (kg) and WC (cm) measured at the level of the umbilicus using an enclosed paper measuring tape. A validation study was performed on 408 participants to compare measures of waist circumference obtained by technicians and by self-report, which showed that the self-reported WC at the level of the umbilicus was highly correlated with the technician-measured WC at the natural WC. The Spearman’s correlation coefficient was 0.87 in men and 0.88 in women [34].

From this information, we calculated change in BW as the difference between measures during 1982–83 and 1987–88 for MONICA, and change in BW and WC during 1993–97 and 1999–2002 for DCH and during 1999–2001 and 2004–06 for INTER99. From this we calculated ∆BW and ∆WC in each cohort by dividing the derived differences with the individual follow-up time in years.

### SNP selection and genotyping

Through review of GWAS, we found 63 SNPs associated to different obesity related phenotypes [8-20,35,36], and 58 of these SNPs were consistently associated with BMI, WC or WHR [8-20]. In the present study, we included SNPs that were available in all three cohorts. Hence, we ended up with a total of 50 SNPs (Additional file 1: Table S1).

In MONICA and DCH, the SNPs were genotyped with the KASPar SNP Genotyping method. In MONICA, they had an average genotyping success rate of 98.3% (minimum 95.8%). In DCH, the average genotyping success rate was 97.8% and 185 replicate samples had a success rate above 98% and an error rate below 0.5%.

Finally, in INTER99 the SNPs were successfully genotyped using either the KASPar SNP Genotyping method, or through Human Cardio-metabo bead chip array (2 SNPs; rs7138803 and rs7647305) using Illumina Hi-Scan technology and GenomeStudio software (http://www.illumina.com/systems/hiscan.ilmn). The average genotyping success rate for the INTER99 study was 96.7% (minimum 94.7).

### Genetic predisposition scores

For each subject, the 50 SNPs were coded 0, 1 or 2 according to number of obesity associated risk alleles. Four different SNP-scores were then calculated as indicators of genetic predisposition: A score of all 50 SNPs (range: 0 to 100), 33 BMI associated SNPs (range: 0 to 66), 6 WC associated SNPs (range: 0 to 12) and 14 WHR associated SNPs (range: 0 to 28), with higher scores indicating higher genetic predisposition to these specific traits.

### Statistical analysis

Linear regression was used to examine the association between dietary ascorbic acid and subsequent ∆BW and ∆WC. First in a crude model with adjustments only for height and baseline measure of outcome, and then in a fully adjusted model where we also included age, sex, smoking status, education level, physical activity, menopausal status and alcohol consumption. Furthermore, to assess associations that were independent of ΔBW, the analysis with ΔWC as outcome was performed both with and without adjustment for concurrent ΔBW. The same procedure was applied using each of the four SNP-scores as the exposure variable. All continuous variables were evaluated by model control (investigating linearity of effects on used outcomes, consistency with a normal distribution and variance homogeneity).

To examine interaction between the four genetic predisposition scores and dietary ascorbic acid in relation to ΔBW or ΔWC, we correspondingly added the SNP-score variables as well as the interaction terms (SNP-score × ascorbic acid). After performing the analysis in the individual cohorts, the results were combined in a meta-analysis. We performed both fixed- and random effect models. The effect-estimates from the individual cohorts were weighted based on the inverses of their variances. Heterogeneity between the studies was assessed by so called Q tests and I^2^-values, where the latter measure indicates the amount of total variation explained by between-study variation [37] and was evaluated according to the following categories: no heterogeneity, I^2^: 0–25%; moderate heterogeneity, I^2^: 25–50%; significant heterogeneity, I^2^: 50–75%; and extreme heterogeneity, I^2^: 75–100%. Since the Q-tests showed no significant heterogeneity and the calculated I^2^-values were all below 50% we only presented results from the fixed effect models. Furthermore, results from the random effect models were almost identical to those from the fixed effect models.

Finally, with exploratory purposes, we performed the analysis of interactions between the 50 individual SNPs and dietary ascorbic acid in relation to ∆BW and ∆WC, with Bonferroni adjustment for multiple testing.

### Supplementary analyses

To further limit the possibility of confounding by energy intake, we performed supplementary analyses where we adjusted for total energy intake. Finally, in DCH we had information about intake of ascorbic acid from supplements, and in MONICA we had information on intake of multivitamins. Hence, in supplementary analyses we also adjusted for these variables.

As described, the DCH participants included in the present study consist of both a sample of BW gainers and a random sample. To maximize the study's statistical power, we decided to include both groups. However, we also performed separate analyses for the two groups.

Furthermore, INTER99 is a multifactorial lifestyle intervention, where the intervention group received a lifestyle counseling talk focusing on smoking, physical activity, diet and alcohol. Hence, in supplementary analyses, we further adjusted the INTER99 analyses for baseline intervention status.

P-values ≤ 0.05 were regarded as statistically significant. All analyses were performed using the statistical software package Stata 12 (StataCorp LP, College Station, Texas, USA; http://www.stata.com).

## Results

### Baseline characteristics

For this study we had information on 1,329 individuals from MONICA, 2,167 from DCH and 4,073 from the INTER99 study. Table 1 shows characteristics of anthropometrics, genetic predisposition scores, intake of dietary ascorbic acid and information on covariates in the three cohorts. In relation to measures of BW and WC, both the baseline values and annual gains were highest among the DCH participants because of the case-cohort design. The median dietary intake of ascorbic acid was 70 mg/day (5-95% percentiles: 23 to 199) among MONICA participants, 93 mg/day (5-95% percentiles: 43 to 207) among DCH participants and 66 mg/day (5-95% percentiles: 25 to 156) among INTER99 participants.

**Table 1:** Ascorbic acid intake, anthropometrics, SNP-scores and covariates in MONICA, DCH and INTER99

	**MONICA**	**DCH**	**INTER99**
N	1,329	2,167	4,073
**Basic variables**			
Ascorbic acid (mg/day)	70 (23 to 199)	93 (43 to 207)	66 (25 to 156)
Baseline age (years)	50.5 (30.6 to 61.1)	53.0 (50.0 to 58.0)	45.1 (34.7 to 59.8)
Sex (% women)	51.6	49.4	51.4
Height (cm)	169 (156 to 184)	171 (158 to 186)	172 (158 to 188)
**BW (kg)**			
Baseline	69.0 (52.0 to 93.0)	77.1 (56.8 to 104.4)	75.5 (55 to 104)
Follow-up	70.0 (51.6 to 94.7)	82.0 (58.0 to 110.0)	76.5 (55.6 to 105.0)
∆BW	0.22 (−1.04 to 1.61)	1.01 (−1.05 to 2.39)	0.20 (−1.41 to 1.75)
**WC (cm)**^**1**^			
Baseline	-	90 (70 to 112)	85 (67 to 107)
Follow-up	-	98 (76 to 121)	88 (69 to 110)
∆WC	-	1.37 (−0.78 to 4.44)	0.55 (−1.27 to 2.46)
**SNP-based variables**^**2**^			
SNP-score (BMI)	29 (23 to 35)	28 (23 to 35)	29 (23 to 34)
SNP-score (WC)	3 (1 to 6)	3 (1 to 6)	3 (1 to 6)
SNP-score (WHR)	14 (10 to 18)	14 (10 to 18)	14 (10 to 18)
SNP-score (complete)	44 (37 to 52)	44 (37 to 51)	44 (36 to 51)
**Adjustment variables**			
Smoking, % Never smokers	27	41.4	40.1
Education, % ≤ Primary school	35.7	30.1	26.2
Physical activity, % most sedentary group	21.4	9.5	11.5
Menopausal status, % postmenopausal	42.4	55.6	27.5
Total energy intake excl. alcohol, (MJ/day)	8.5 (4.8 to 13.9)	8.2 (4.9 to 13.4)	9.0 (4.9 to 15.1)
Alcohol (MJ/day)	0.4 (0 to 1.8)	0.4 (0 to 1.9)	0.3 (0 to 1.5)

*Abbreviations*: *BW* body weight, *WC* waist circumference, *∆BW* annual weight change, *∆WC* annual WC change.

^1^DCH: n = 2,165 on baseline WC, n = 2,130 on follow-up WC and n = 2,128 on ∆WC. INTER99: n = 4,067 on baseline WC, n = 3,540 on follow-up WC and n = 3,536 on ∆WC.

^2^Sum of BMI, WC or WHR associated risk-alleles. In MONICA information was available in n = 989 on BMI SNP-score, n = 1,250 on WC SNP-score, n = 1,185 on WHR SNP-score and n = 878 on complete SNP-score. In DCH n = 1,438 on BMI SNP-score, n = 1,805 on WC SNP-score, n = 1,624 on WHR SNP-score and n = 1,247 on complete SNP-score. In INTER99 n = 2,511 on BMI SNP-score, n = 3,381 on WC SNP-score, n = 3,264 on WHR SNP-score, n = 2,082 on complete score.

Results presented as median (5–95 percentiles) unless otherwise stated.

The genetic predisposition scores were nearly identical in terms of median and 5-95% percentiles in all three cohorts. Since there was not complete information on all the included SNPs, the sample size varies slightly depending on what SNP-score we included. The score consisting of all 50 SNPs (n: MONICA = 878, DCH = 1,247, INTER99 = 2,082), the score of 33 BMI associated SNPs (n: MONICA = 989, DCH = 1,438, INTER99 = 2,511), the score of 6 WC associated SNPs (n: MONICA = 1,250, DCH = 1,805, INTER99 = 3,381) and the score of 14 WHR associated SNPs (n: MONICA = 1,185, DCH = 1,624, INTER99 = 3,264).

### Dietary ascorbic acid in relation to change in weight and waist circumference

The cross-sectional associations between dietary ascorbic acid and BW or WC are presented in Additional file 1: Table S2. We did not find a statistically significant association between baseline ascorbic acid and BW. However, after adjusting for potential confounders we found a significantly lower WC of −0.64 cm (p = 0.03, CI −1.22 to −0.05) per 100 mg/day ascorbic acid.

The association between ascorbic acid intake and ∆BW as well as ∆WC is presented in Table 2 for both the crude and the adjusted models. The result from the meta-analysis of the crude model showed a ∆BW of −0.048 kg (P = 0.03, CI: −0.092 to −0.004) per 100 mg/day higher ascorbic acid. However, after adjusting for potential confounders, the result from the meta-analysis was not statistically significant with a ∆BW of −0.014 (P = 0.54, CI: −0.059 to 0.031). Likewise, we found no statistically significant association between dietary ascorbic acid and ∆WC.

**Table 2:** Annual change in body weight (kg) and waist circumference (cm) pr. 100 mg/day higher ascorbic acid intake in the three cohorts

	**N**	**Crude β (95% CI)**^**1**^	**% weight**^**3**^	**Adjusted β (95% CI)**^**2**^	**% weight**
**Annual weight change (kg)**					
MONICA	1,329	−0.024 (−0.097 to 0.049)	36.38	−0.018 (−0.093 to 0.057)	36.84
DCH	2,167	−0.103 (−0.192 to −0.015)	24.71	−0.082 (−0.172 to 0.009)	25.11
INTER99	4,073	−0.035 (−0.106 to 0.035)	38.91	0.035 (−0.039 to 0.108)	38.05
Overall	7,569	−0.048 (−0.092 to −0.004)	100	−0.014 (−0.059 to 0.031)	100
**Annual waist change (cm)**					
DCH	2,128	−0.119 (−0.246 to 0.007)	32.82	−0.111 (−0.219 to 0.003)	26.28
INTER99	3,536	−0.010 (−0.098 to 0.079)	67.12	0.010 (−0.074 to 0.055)	73.72
Overall	5,664	−0.046 (−0.118 to 0.027)	100	−0.034 (−0.089 to 0.021)	100

^1^Adjusted for baseline outcome and height.

^2^Adjusted for baseline outcome, height, sex, age, smoking status, alcohol consumption, physical activity, education and menopausal status for women. Analysis with change in waist circumference adjusted for concurrent weight change.

^3^Estimates were calculated in MONICA, DCH and INTER99 using linear regression and meta-analysed using a fixed effect meta-analysis approach. Individual cohorts were weighted based on the inverses of their variances (% weight).

### Genetic predisposition scores in relation to weight and waist circumference

The “main effects” of the four SNP-scores have been published elsewhere [38]. Since the scores are based on SNPs identified through cross sectional GWAS, we generally observed statistically significant associations with BW and WC at baseline, with the exception of the WHR-score. However, when analyzing the associations between our four SNP-scores and subsequent changes in BW and WC, neither the crude nor the adjusted models showed any statistically significant associations [38].

### Interaction between genetic predisposition scores and dietary ascorbic acid

**Figure 1:**
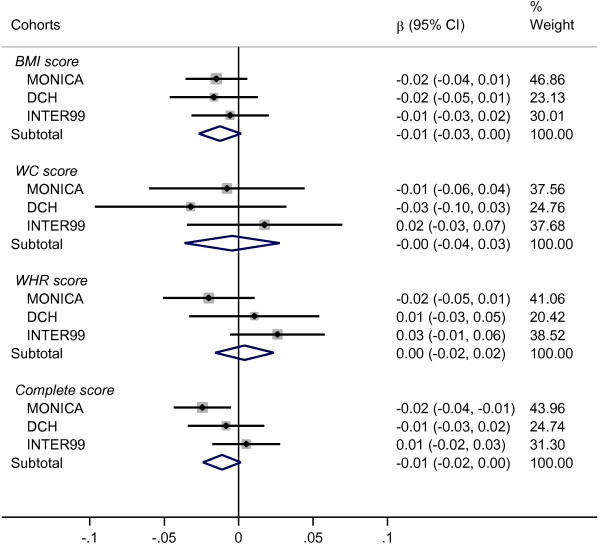
**Interaction between genetic predisposition scores and dietary ascorbic acid in relation to change in body weight.** Abbreviations: BMI score, sum of body mass index associated risk-alleles; WC score, sum of waist circumference associated risk-alleles; WHR score, sum of waist-hip ratio associated risk-alleles; Complete score, sum of SNP associated to all three phenotypes. Results presented as annual weight change (kg/year) effect-modification for each additional risk-allele per 100 mg/day higher ascorbic acid intake. The study-specific SNP-score × ascorbic acid interactions were calculated using linear regression and corresponding meta-analysis results were derived using a fixed effect approach, where the effect-estimates where weighted by the inverses of their variances (% weight). The results were adjusted for baseline measure of body weight, height, sex, age, smoking status, alcohol consumption, physical activity, education and menopausal status for women.

In Figure 1, the results on SNP-score × ascorbic acid interactions are presented in relation to ∆BW. The figure shows the association between ascorbic acid and ∆BW per additional risk allele from each of the four SNP-scores. In the meta-analysis, we found no significant interactions between any of the four SNP-scores and dietary ascorbic acid in relation to the subsequent change in BW. However, we did find a tendency towards an interaction between the score of 33 BMI associated SNPs and ascorbic acid in relation to ∆BW. Each additional risk allele from this score was associated with an average ∆BW effect-modification of −0.013 kg/year (P = 0.08, 95% CI: −0.027 to 0.002) per 100 mg/day higher ascorbic acid intake.

To further investigate this tendency, we looked at the association between ascorbic acid and ∆BW in tertiles of the BMI associated SNP-score (Additional file 1: Figure S1). Using this approach, we found no statistically significant association between ascorbic acid and ∆BW in any of the tertiles.

**Figure 2:**
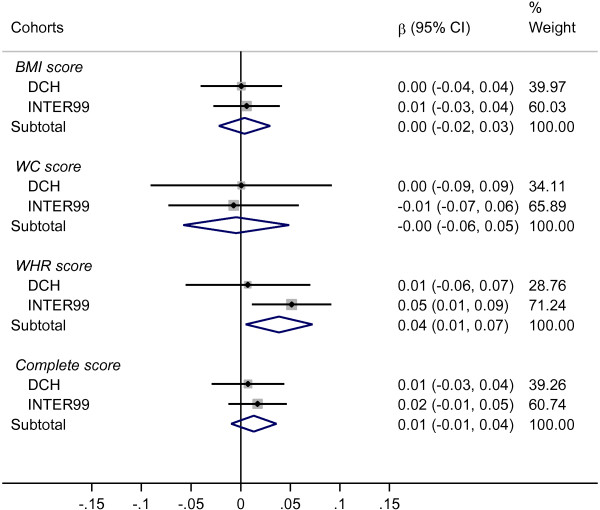
**Interaction between genetic predisposition scores and dietary ascorbic acid in relation to change in waist circumference.** Abbreviations: BMI score, sum of body mass index associated risk-alleles; WC score, sum of waist circumference associated risk-alleles; WHR score, sum of waist-hip ratio associated risk-alleles; Complete score, sum of SNP associated to all three phenotypes. Results presented as annual change in waist circumference (cm/year) for each additional risk-allele per 100 mg/day higher ascorbic acid intake. The study-specific SNP-score × ascorbic acid interactions were calculated using linear regression and corresponding meta-analysis results where derived using a fixed effect approach, were the effect-estimates were weighted by the inverses of their variances (% weight). The results were adjusted for baseline measure of waist circumference, height, sex, age, smoking status, alcohol consumption, physical activity, education and menopausal status for women.

Figure 2 shows the results on SNP-score × ascorbic acid interactions in relation to ∆WC. In the meta-analysis, we found no statistically significant interactions between the scores of BMI or WC associated SNPs and dietary ascorbic acid. However, we found an interaction between the score of 14 WHR associated SNPs and ascorbic acid. In the meta-analysis, each additional risk allele was associated with an average ∆WC effect-modification of 0.039 cm/year (P = 0.02, 95% CI: 0.005 to 0.073) per 100 mg/day higher ascorbic acid intake. After adjusting for ∆BW, the tendency in the direction of the association persisted, but was not statistically significant (Additional file 1: Figure S2).

We further looked at the association between ascorbic acid and ∆WC in tertiles of the WHR associated SNP-score, both with and without adjustment for ∆BW (Additional file 1: Figure S3). Using this procedure, we found no statistically significant association between ascorbic acid and ∆WC in any of the tertiles.

Finally, we investigated interaction between the individual SNPs and ascorbic acid in relation to ΔBW (Additional file 1: Table S3) and ΔWC (Additional file 1: Table S4). After adjusting for multiple testing none of the results were statistically significant.

### Supplementary analyses

Additional adjustment for total energy had virtually no influence on the observed associations. Likewise, further adjustment for ascorbic acid from supplements in DCH, and multivitamin supplements in MONICA, had negligible influence on the observed associations.

As described, the sample from the DCH cohort consists of a group of weight gainers and a group of randomly selected participants. Hence, we further performed analyses separately for the two groups. However, neither results on main effect of ascorbic acid in relation to change in ∆BW or ∆WC nor results on interaction with genetic predispositions scores, showed any statistically significant difference between the two groups.

Furthermore, in INTER99 we adjusted for baseline intervention status, and this did not change the direction or the statistical significance of the reported results.

## Discussion

Using data from three cohorts of 7,569 Danish men and women, we found no statistically significant association between ascorbic acid intake and ∆BW or ∆WC in general. Likewise, although the four SNP-scores generally showed strong associations to baseline anthropometry they did not associate to change in these measures. We did, however, find a statistically significant interaction between a score of 14 WHR-associated SNPs and dietary ascorbic acid in relation to ∆WC, suggesting that dietary ascorbic acid may be associated to a higher gain in WC among people genetically predisposed to a high WHR. However, the WHR-score × ascorbic acid interactions in relation to ∆WC was only borderline significant, after adjustment for concurrent ∆BW. Furthermore, we found a tendency towards an interaction between a score of 33 BMI-associated SNPs and dietary ascorbic acid. This interaction suggested that intake of ascorbic acid may be associated with a lower subsequent BW gain among people who are genetically predisposed to a high BMI. Finally, we found no statistically significant interactions in relation to ∆BW using scores of SNPs associated with WC, WHR or the sum of all 50 obesity associated SNPs. Likewise, we found no statistically significant interactions in relation to ∆WC using scores of BMI, WC or the sum of all 50 obesity associated SNPs.

The main strengths of our study include the detailed measures of dietary information collected either with 7-day food records or FFQs along with repeated measures of BW and WC as well as information on potential confounders. Finally, we had genetic information on 50 SNPs selected based on their consistent cross-sectional association with obesity related traits, allowing us to calculate genetic predisposition scores. Despite the fact that the sum of these variants only explain very little of the total variation in these measures of adiposity (<5%) this has become one of the favored methods to study gene × environment interactions in relation to obesity [39,40].

However, our study also has some limitations. Regardless of the method used to collect dietary data, these methods are generally subject to inaccuracies. In addition, dietary content of ascorbic acid is highly dependent on how the food has been prepared [41] and stored [42], and although preparation of the food has been taken into account when nutrient calculations were performed, this could affect the accuracy of our exposure measure. Hence, measurement error related to dietary intake could have biased the results towards null and led to wider confidence intervals.

Furthermore, dietary intake of ascorbic acid is not necessarily a good indicator of the biological status, and therefore measures of serum ascorbic acid would have strengthened the study. Also, we do not know with certainty whether the results of our study are directly caused by ascorbic acid or other common factors of a diet with a high content of this specific nutrient. Hence, dietary ascorbic acid may be an indicator of a particular dietary pattern and lifestyle, and it is possible that some of these factors account for the observed associations and not the actual nutrient per se. It has been suggested that consumption of fruit and vegetables, which is the primary source of ascorbic acid, is weakly associated to BW loss [43]. Hence, associations observed in our study could also be due to other characteristics of this food group, such as a high content of dietary fiber and flavonoids. Furthermore, contrary to our expectations, dietary ascorbic acid appeared to be associated to a higher WC gain among those who were genetically predisposed to a high WHR. In this context, an important argument is that we consume ascorbic acid from a wide variety of foods. Hence, a possible explanation for our results could be that the genetically predisposed prefer to consume ascorbic acid from other dietary sources than the less predisposed. Hence, we do not know whether our results actually indicate a different effect of ascorbic acid on changes in WC depending on the genetic predisposition, or whether the results are an expression of different dietary preferences between genetic subgroups. Thus, the observed results should be interpreted with caution.

Finally, studies of specific dietary nutrients, as well as studies on genetic variants in relation to obesity, have generally shown relatively weak associations. Hence, it is likely that the size of any SNP × calcium interactions is also minor. Thus, a population of 7,569 individuals might be too small to discover potential SNP × calcium interactions. As a result, it is possible that we have overlooked some associations due to lack of statistical power. However, the interaction estimates generally had very narrow confidence intervals, suggesting it is unlikely that we have overlooked large associations. In addition, the size of the statistically significant interaction observed in our study was very small, indicating that we had sufficient statistical power to measure an association so small that any public health relevance is doubtful.

The majority of published studies examining the associations between ascorbic acid and BW or WC have been cross-sectional [2-4,44] and although these studies find that low ascorbic acid status is associated with higher BW or WC they do not shed light on a possible causal relationship. However, Naylor et al. (1985) published results from a double blind placebo controlled trial of 38 obese subjects. In this study, they demonstrated that supplementation with 3 g of ascorbic acid per day increased weight loss compared to placebo during a 6-week period [5]. Moreover, studies have shown that mice fed on a high fat diet supplemented with ascorbic acid gained less adipose tissue than their non-supplemented controls [45,46].

In our study, we were unable to find a significant association between dietary ascorbic acid and subsequent change in BW. One possible explanation for the different results in our study and these previous studies is that supplementation with high doses of ascorbic acid may lead to other biological effects than dietary intake. In addition, due to a higher oxidative stress status, it is possible that the relationship is primarily present among obese individuals or people eating a high fat diet [45].

It has been proposed that ascorbic acid may decrease BW and WC through a contribution to production of the amino acid carnitine, which is required in the oxidation of fatty acids [7]. Whether these or other biological mechanisms depend on genetic predisposition to high BMI, WHR or WC is speculative due to the limited knowledge about the biological implications of the included genetic variants.

Finally, it should be taken into account that the participants included in our study were not necessarily representative of the general Danish adult population. Especially, participants from the DCH sample of which half was a selected group of weight gainers. Furthermore, participants in the three cohorts have a healthier lifestyle than the general Danish population. Consequently, generalizations from our results should be made with caution.

## Conclusions

Our study does not suggest that dietary ascorbic acid is generally related to ∆BW or ∆WC. However, a diet high in ascorbic acid may be associated with a slightly higher WC gain among people genetically predisposed to a high WHR. Nevertheless, the associations were generally weak and inconsistent. Hence, further replications are needed before final conclusions regarding these associations can be reached.

## Abbreviations

BW: Body weight; CI: Confidence interval; DCH: Diet cancer and health study; FFQ: Food frequency questionnaire; GWAS: Genome-wide association studies; SNP: Single-nucleotide polymorphism; WC: Waist circumference; WHR: Waist-hip ratio; ∆BW: Annual change in body weight; ∆WC: Annual change in waist circumference.

## Competing interests

None of the authors had a financial or personal conflict of interest.

## Authors’ contributions

The authors’ responsibilities were as follows – The present study was conceived by TIAS, BLH and SCL and designed by SCL, LÄ, BLH and TIAS. SCL wrote the manuscript, prepared tables and figures, and conducted the statistical analyses under the supervision of TIAS, BLH and LÄ. TS, AL, BLH, LLNH, UT, NR, KO, AT, JH, TSA, OP, and TH helped acquire data, interpret the results and provided comments on the manuscript. All authors read and approved the final manuscript.

## Supplementary Material

Additional file 1: Table S1 Information on the 50 SNP's included in this study. The individual SNPs are sorted by refSNP (rs) number and grouped according to their associated trait. **Table S2.** Baseline body weight (kg) and waist circumference (cm) pr. 100 mg/day higher ascorbic acid intake in the three cohorts. Results are presented both from the crude and the adjusted models. **Table S3.** SNP × ascorbic acid interaction in relation to annual change in body weight (kg/year) per 100 mg/day higher ascorbic acid intake in MONICA, DCH and INTER99 (β and p-value). The results are sorted by refSNP (rs) number and grouped according to their associated trait. **Table S4.** SNP × ascorbic acid interaction in relation to annual change in waist circumference (cm/year) per 100 mg/day higher ascorbic acid intake in MONICA, DCH and INTER99 (β and p-value). The results are sorted by refSNP (rs) number and grouped according to their associated trait. **Figure S1.** Annual change in body weight (kg/year) per 100 mg/day higher ascorbic acid intake in tertiles of the BMI associated SNP score. **Figure S2.** Interaction between genetic predisposition scores and dietary ascorbic acid in relation to change in waist circumference adjusted for concurrent change in body weight. **Figure S3.** Annual change in waist circumference (cm/year) per 100 mg/day higher ascorbic acid intake in tertiles of the WHR associated SNP score. Click here for file
