# Supplementary material for: Dietary ascorbic acid and subsequent change in body weight and waist circumference: associations may depend on genetic predisposition to obesity - a prospective study of three independent cohorts
Source: Nutr J. 2014 May 3;13:43. doi: 10.1186/1475-2891-13-43 (PMC4024624; doi:10.1186/1475-2891-13-43)
Supplement: Additional file 1: Table S1 — Information on the 50 SNP's included in this study. The individual SNPs are sorted by refSNP (rs) number and grouped according to their associated trait. Table S2. Baseline body weight (kg) and waist circumference (cm) pr. 100 mg/day higher ascorbic acid intake in the three cohorts. Results are presented both from the crude and the adjusted models. Table S3. SNP × ascorbic acid interaction in relation to annual change in body weight (kg/year) per 100 mg/day higher ascorbic acid intake in MONICA, DCH and INTER99 (β and p-value). The results are sorted by refSNP (rs) number and grouped according to their associated trait. Table S4. SNP × ascorbic acid interaction in relation to annual change in waist circumference (cm/year) per 100 mg/day higher ascorbic acid intake in MONICA, DCH and INTER99 (β and p-value). The results are sorted by refSNP (rs) number and grouped according to their associated trait. Figure S1. Annual change in body weight (kg/year) per 100 mg/day higher ascorbic acid intake in tertiles of the BMI associated SNP score. Figure S2. Interaction between genetic predisposition scores and dietary ascorbic acid in relation to change in waist circumference adjusted for concurrent change in body weight. Figure S3. Annual change in waist circumference (cm/year) per 100 mg/day higher ascorbic acid intake in tertiles of the WHR associated SNP score. [file 1475-2891-13-43-S1.docx]

| **Table S1: Information on the 50 SNP´s included in this study. The individual SNPs are sorted by refSNP (rs) number and grouped according to their associated trait** | | | | |
| --- | --- | --- | --- | --- |
| **Trait** | **SNP** | **Nearest gene** | **Risk allele** | **Other allele** |
|  |  |  |  |  |
| BMI | rs10508503 | PTER | C | T |
| BMI | rs10838738 | MTCH2 | G | A |
| BMI | rs10938397 | GNPDA2 | G | A |
| BMI | rs10968576 | LRRN6C | G | A |
| BMI | rs11847697 | PRKD1 | T | C |
| BMI | rs12444979 | GPRC5B | C | T |
| BMI | rs13107325 | SLC39A8 | T | C |
| BMI | rs1424233 | MAF | A | G |
| BMI | rs1514175 | TNNI3K | T | C |
| BMI | rs1555543 | PTBP2 | C | A |
| BMI | rs17782313 | MC4R | C | T |
| BMI | rs1805081 | NPC1 | A | G |
| BMI | rs206936 | NUDT3 | G | A |
| BMI | rs2112347 | FLJ35779 | T | G |
| BMI | rs2241423 | MAP2K5 | G | A |
| BMI | rs2287019 | QPCTL | C | T |
| BMI | rs2568958 | NEGR1 | A | G |
| BMI | rs2890652 | LRP1B | C | T |
| BMI | rs29941 | KCTD15 | G | A |
| BMI | rs3810291 | TMEM160 | A | G |
| BMI | rs4712652 | LINC00340 | A | G |
| BMI | rs4771122 | MTIF3 | G | A |
| BMI | rs4929949 | RPL27A | C | T |
| BMI | rs543874 | SEC16B | G | A |
| BMI | rs6013029 | CTNNBL1 | T | G |
| BMI | rs6232 | PCSK1 | G | A |
| BMI | rs6602024 | PFKP | A | G |
| BMI | rs713586 | RBJ | C | T |
| BMI | rs7647305 | SFRS10 | C | T |
| BMI | rs9939609 | FTO | A | T |
| BMI/WC | rs10146997 | NRXN3 | G | A |
| BMI/WC | rs1121980 | FTO | A | G |
| BMI/WC | rs7138803 | FAIM2 | A | G |
| WC | rs12970134 | MC4R | A | G |
| WC | rs545854 | MSRA | G | C |
| WC | rs987237 | TFAPB2 | G | A |
| WHR | rs1011731 | DNM3-PIGC | C | T |
| WHR | rs10195252 | GRB14 | T | C |
| WHR | rs1055144 | NFE2L3 | A | G |
| WHR | rs1294421 | LY86 | G | T |
| WHR | rs1443512 | HOXC13 | A | C |
| WHR | rs2605100 | LYPLAL1 | G | A |
| WHR | rs4823006 | ZNRF3-KREMEN1 | A | G |
| WHR | rs6784615 | NISCH-STAB1 | T | C |
| WHR | rs6795735 | ADAMTS9 | C | T |
| WHR | rs6861681 | CPEB4 | A | G |
| WHR | rs6905288 | VEGFA | A | G |
| WHR | rs718314 | ITPR2-SSPN | C | T |
| WHR | rs9491696 | RSPO3 | G | C |
| WHR | rs984222 | TBX15-WARS2 | G | C |
| Abbreviations: BMI, body mass index; WC, waist circumference; WHR, Waist-hip ratio. | | | | |

| **Table S2: Baseline body weight (kg) and waist circumference (cm) pr. 100 mg/day higher ascorbic acid intake in the three cohorts. Results are presented both from the crude and the adjusted models** | | | | | |
| --- | --- | --- | --- | --- | --- |
|  | N | Crude β (95% CI)^1^ | % weight ^3^ | Adjusted β (95% CI) ^2^ | % weight |
| **Body weight (kg)** |  |  |  |  |  |
| MONICA | 1,329 | 0.38 (-0.53 to 1.30) | 34.55 | 0.61 (-0.31 to 1.53) | 36.41 |
| DCH | 2,167 | -1.37 (-2.35 to -0.39) | 30.29 | -0.26 (-1.32 to 0.80) | 27.62 |
| INTER99 | 4,073 | -0.19 (-1.10 to 0.72) | 35.16 | -0.10 (-1.03 to 0.83) | 35.97 |
| Overall | 7,569 | -0.35 (-0.89 to 0.18) | 100 | 0.12 (-0.44 to 0.67) | 100 |
| **Waist circumference (cm)** |  |  |  |  |  |
| DCH | 2,128 | -2.45 (-3.37 to -1.53) | 44.31 | -1.05 (-1.92 to -0.17) | 44.30 |
| INTER99 | 3,536 | -1.12 (-1.94 to -0.30) | 55.69 | -0.31 (-1.09 to 0.47) | 55.70 |
| Overall | 5,664 | -1.71 (-2.32 to -1.10) | 100 | -0.64 (-1.22 to -0.05) | 100 |
| *^1^Adjusted for height.*  *^2^Adjusted for height, sex, age, smoking status, alcohol consumption, leisure time physical activity, education and menopausal status for women.*  *^3^ Estimates were calculated in MONICA, DCH and INTER99 using linear regression and meta-analysed using a fixed effect meta-analysis approach. Individual cohorts were weighted based on the inverses of their variances (% weight).* | | | | | |

| **Table S3: SNP×** **ascorbic acid interaction in relation to annual change in body weight (kg/year) per 100 mg/day higher ascorbic acid intake in MONICA, DCH and INTER99 (β and p-value). The results are sorted by refSNP (rs) number and grouped according to their associated trait** | | | | | | | |
| --- | --- | --- | --- | --- | --- | --- | --- |
| **Trait** | **SNP** | **MONICA** | | **DCH** | | **INTER99** | |
|  |  | **(N=1,329)** | | **(N=2,167)** | | **(N=4,073)** | |
|  |  | **β** | **P-value** | **β** | **P-value** | **β** | **P-value** |
| BMI | rs10508503 | 0.125 | 0.222 | -0.208 | 0.125 | -0.011 | 0.909 |
| BMI | rs10838738 | 0.016 | 0.779 | 0.077 | 0.271 | 0.057 | 0.296 |
| BMI | rs10938397 | -0.142 | 0.010 | -0.118 | 0.086 | -0.013 | 0.796 |
| BMI | rs10968576 | 0.017 | 0.766 | 0.050 | 0.504 | -0.031 | 0.589 |
| BMI | rs11847697 | -0.151 | 0.446 | -0.187 | 0.283 | -0.112 | 0.412 |
| BMI | rs12444979 | 0.027 | 0.741 | -0.014 | 0.890 | -0.085 | 0.259 |
| BMI | rs13107325 | 0.063 | 0.667 | 0.117 | 0.526 | -0.015 | 0.902 |
| BMI | rs1424233 | 0.065 | 0.268 | -0.150 | 0.030 | 0.048 | 0.344 |
| BMI | rs1514175 | -0.074 | 0.168 | -0.105 | 0.132 | 0.031 | 0.543 |
| BMI | rs1555543 | 0.005 | 0.922 | -0.130 | 0.066 | -0.021 | 0.690 |
| BMI | rs17782313 | -0.018 | 0.773 | -0.008 | 0.923 | 0.019 | 0.748 |
| BMI | rs1805081 | -0.034 | 0.528 | 0.163 | 0.025 | -0.026 | 0.628 |
| BMI | rs206936 | 0.017 | 0.775 | 0.009 | 0.920 | -0.041 | 0.542 |
| BMI | rs2112347 | -0.080 | 0.129 | 0.049 | 0.515 | 0.019 | 0.714 |
| BMI | rs2241423 | 0.020 | 0.753 | 0.208 | 0.009 | -0.078 | 0.201 |
| BMI | rs2287019 | -0.077 | 0.268 | 0.034 | 0.693 | 0.050 | 0.433 |
| BMI | rs2568958 | 0.024 | 0.683 | 0.043 | 0.522 | 0.043 | 0.427 |
| BMI | rs2890652 | -0.005 | 0.941 | -0.016 | 0.871 | 0.122 | 0.069 |
| BMI | rs29941 | -0.032 | 0.611 | 0.037 | 0.588 | -0.078 | 0.185 |
| BMI | rs3810291 | 0.025 | 0.666 | -0.135 | 0.057 | -0.042 | 0.450 |
| BMI | rs4712652 | -0.030 | 0.591 | -0.080 | 0.243 | 0.062 | 0.228 |
| BMI | rs4771122 | -0.035 | 0.574 | 0.029 | 0.712 | 0.007 | 0.908 |
| BMI | rs4929949 | -0.016 | 0.770 | -0.118 | 0.147 | -0.013 | 0.804 |
| BMI | rs543874 | 0.056 | 0.418 | -0.007 | 0.938 | 0.001 | 0.989 |
| BMI | rs6013029 | -0.163 | 0.122 | -0.163 | 0.250 | 0.071 | 0.551 |
| BMI | rs6232 | 0.044 | 0.724 | 0.261 | 0.097 | -0.014 | 0.896 |
| BMI | rs6602024 | -0.014 | 0.850 | -0.319 | 0.005 | -0.095 | 0.268 |
| BMI | rs713586 | 0.027 | 0.604 | -0.140 | 0.051 | 0.107 | 0.036 |
| BMI | rs7647305 | -0.032 | 0.636 | 0.029 | 0.740 | -0.018 | 0.801 |
| BMI | rs9939609 | 0.004 | 0.939 | -0.020 | 0.753 | -0.067 | 0.199 |
| BMI/WC | rs10146997 | -0.069 | 0.310 | -0.078 | 0.366 | 0.012 | 0.847 |
| BMI/WC | rs1121980 | 0.004 | 0.932 | -0.014 | 0.840 | 0.001 | 0.984 |
| BMI/WC | rs7138803 | 0.017 | 0.765 | -0.053 | 0.435 | 0.080 | 0.156 |
| WC | rs12970134 | 0.016 | 0.784 | -0.044 | 0.566 | -0.011 | 0.850 |
| WC | rs545854 | -0.012 | 0.864 | 0.024 | 0.795 | -0.016 | 0.821 |
| WC | rs987237 | -0.033 | 0.654 | -0.024 | 0.794 | -0.005 | 0.942 |
| WHR | rs1011731 | -0.047 | 0.360 | 0.082 | 0.285 | 0.033 | 0.529 |
| WHR | rs10195252 | -0.001 | 0.985 | 0.053 | 0.464 | -0.066 | 0.205 |
| WHR | rs1055144 | 0.023 | 0.753 | 0.072 | 0.421 | -0.125 | 0.065 |
| WHR | rs1294421 | 0.048 | 0.369 | -0.048 | 0.482 | -0.035 | 0.529 |
| WHR | rs1443512 | -0.031 | 0.636 | 0.001 | 0.994 | 0.144 | 0.020 |
| WHR | rs2605100 | -0.045 | 0.417 | 0.079 | 0.293 | 0.128 | 0.026 |
| WHR | rs4823006 | 0.035 | 0.529 | 0.027 | 0.690 | 0.000 | 0.995 |
| WHR | rs6784615 | 0.100 | 0.447 | 0.340 | 0.027 | 0.171 | 0.102 |
| WHR | rs6795735 | -0.044 | 0.447 | -0.052 | 0.490 | 0.091 | 0.083 |
| WHR | rs6861681 | 0.017 | 0.759 | 0.075 | 0.282 | 0.171 | 0.002 |
| WHR | rs6905288 | 0.003 | 0.957 | -0.042 | 0.544 | -0.056 | 0.273 |
| WHR | rs718314 | -0.033 | 0.600 | -0.134 | 0.099 | -0.014 | 0.806 |
| WHR | rs9491696 | 0.015 | 0.778 | 0.047 | 0.506 | -0.051 | 0.323 |
| WHR | rs984222 | -0.013 | 0.807 | 0.072 | 0.315 | 0.008 | 0.878 |
| Abbreviations: BMI, body mass index; WC, waist circumference; WHR, waist-hip ratio.  Adjusted for baseline BW, height, sex, age, smoking status, alcohol consumption, physical activity, education and menopausal status for women. | | | | | | | |

| **Table S4: SNP×** **ascorbic acid interaction in relation to annual change in waist circumference (cm/year) per 100 mg/day higher ascorbic acid intake in MONICA, DCH and INTER99 (β and p-value). The results are sorted by refSNP (rs) number and grouped according to their associated trait** | | | | | |
| --- | --- | --- | --- | --- | --- |
| **Trait** | **SNP** | **DCH** | | **INTER99** | |
|  |  | **(2,128)** | | **(3,536)** | |
|  |  | **β** | **P-value** | **β** | **P-value** |
| BMI | rs10508503 | -0.406 | 0.034 | -0.011 | 0.924 |
| BMI | rs10838738 | 0.154 | 0.119 | 0.064 | 0.340 |
| BMI | rs10938397 | -0.082 | 0.399 | -0.041 | 0.534 |
| BMI | rs10968576 | 0.049 | 0.643 | -0.064 | 0.362 |
| BMI | rs11847697 | 0.127 | 0.607 | -0.257 | 0.135 |
| BMI | rs12444979 | -0.133 | 0.357 | -0.066 | 0.480 |
| BMI | rs13107325 | 0.293 | 0.260 | 0.112 | 0.446 |
| BMI | rs1424233 | -0.057 | 0.561 | 0.031 | 0.622 |
| BMI | rs1514175 | -0.210 | 0.033 | 0.034 | 0.588 |
| BMI | rs1555543 | -0.014 | 0.886 | 0.035 | 0.597 |
| BMI | rs17782313 | 0.074 | 0.512 | 0.011 | 0.889 |
| BMI | rs1805081 | 0.041 | 0.693 | -0.055 | 0.415 |
| BMI | rs206936 | -0.058 | 0.637 | -0.065 | 0.445 |
| BMI | rs2112347 | 0.078 | 0.468 | 0.001 | 0.993 |
| BMI | rs2241423 | 0.215 | 0.057 | -0.097 | 0.205 |
| BMI | rs2287019 | -0.211 | 0.082 | 0.078 | 0.323 |
| BMI | rs2568958 | 0.058 | 0.546 | -0.022 | 0.742 |
| BMI | rs2890652 | 0.174 | 0.204 | 0.157 | 0.056 |
| BMI | rs29941 | -0.001 | 0.991 | -0.092 | 0.217 |
| BMI | rs3810291 | -0.204 | 0.044 | -0.070 | 0.324 |
| BMI | rs4712652 | -0.055 | 0.568 | 0.100 | 0.119 |
| BMI | rs4771122 | 0.076 | 0.489 | 0.022 | 0.766 |
| BMI | rs4929949 | -0.027 | 0.783 | 0.011 | 0.865 |
| BMI | rs543874 | 0.094 | 0.456 | 0.057 | 0.509 |
| BMI | rs6013029 | -0.068 | 0.735 | 0.093 | 0.519 |
| BMI | rs6232 | 0.223 | 0.311 | -0.085 | 0.525 |
| BMI | rs6602024 | -0.161 | 0.317 | -0.107 | 0.311 |
| BMI | rs713586 | -0.090 | 0.375 | 0.170 | 0.010 |
| BMI | rs7647305 | 0.138 | 0.266 | -0.088 | 0.312 |
| BMI | rs9939609 | 0.092 | 0.309 | -0.029 | 0.659 |
| BMI/WC | rs10146997 | -0.079 | 0.513 | -0.069 | 0.400 |
| BMI/WC | rs1121980 | 0.109 | 0.272 | 0.051 | 0.461 |
| BMI/WC | rs7138803 | -0.011 | 0.906 | -0.030 | 0.676 |
| WC | rs12970134 | 0.044 | 0.692 | -0.027 | 0.717 |
| WC | rs545854 | 0.077 | 0.554 | -0.098 | 0.271 |
| WC | rs987237 | -0.310 | 0.017 | -0.003 | 0.975 |
| WHR | rs1011731 | -0.217 | 0.044 | 0.052 | 0.430 |
| WHR | rs10195252 | 0.131 | 0.205 | 0.047 | 0.480 |
| WHR | rs1055144 | 0.159 | 0.210 | -0.105 | 0.217 |
| WHR | rs1294421 | -0.003 | 0.978 | 0.029 | 0.671 |
| WHR | rs1443512 | 0.021 | 0.849 | 0.095 | 0.219 |
| WHR | rs2605100 | -0.095 | 0.376 | 0.154 | 0.031 |
| WHR | rs4823006 | 0.022 | 0.819 | 0.050 | 0.436 |
| WHR | rs6784615 | 0.144 | 0.516 | 0.123 | 0.426 |
| WHR | rs6795735 | -0.148 | 0.158 | 0.124 | 0.057 |
| WHR | rs6861681 | -0.110 | 0.268 | 0.130 | 0.059 |
| WHR | rs6905288 | -0.047 | 0.631 | -0.014 | 0.830 |
| WHR | rs718314 | -0.017 | 0.880 | 0.040 | 0.588 |
| WHR | rs9491696 | 0.077 | 0.442 | -0.011 | 0.869 |
| WHR | rs984222 | 0.020 | 0.845 | -0.060 | 0.388 |
| Abbreviations: BMI, body mass index; WC, waist circumference; WHR, waist-hip ratio.  Adjusted for baseline WC, height, sex, age, smoking status, alcohol consumption, physical activity, education and menopausal status for women. | | | | | |

**Figure S1: Annual change in body weight (kg/year) per 100 mg/day higher ascorbic acid intake in tertiles of the BMI associated SNP score**

*Abbreviations: BMI score, sum of body mass index associated risk-alleles*

*Estimates were calculated in MONICA, DCH and INTER99 using linear regression and meta-analyzed using a fixed effect approach.*

*The results were adjusted for baseline measure of body weight, height, sex, smoking status,* *alcohol consumption, physical activity, education and menopausal status for women.*

*Participants per tertile (range of risk alleles): T1 N=1,846 (15-27), T2 n=1,614 (28-29) and T3 n=1,478 (31-42).*

**Figure S2: Interaction between genetic predisposition scores and dietary ascorbic acid in relation to change in waist circumference adjusted for concurrent change in body weight**

*Abbreviations: BMI score, sum of body mass index associated risk-alleles; WC score, sum of waist circumference associated risk-alleles; WHR score, sum of waist-hip ratio associated risk-alleles; Complete score, sum of SNP associated to all three phenotypes.*

*Results presented as annual change in waist circumference (cm/year) for each additional risk-allele per 100 mg/day higher ascorbic acid intake.*

*The study-specific SNP-score×ascorbic acid interactions were calculated using linear regression and corresponding meta-analysis results were derived using a fixed effect approach, were the effect-estimates were weighted by the inverses of their variances (% weight).*

*The results were adjusted for baseline measure of waist circumference, height, sex, age, smoking status, alcohol consumption, physical activity, education and menopausal status for women.*

**Figure S3: Annual change in waist circumference (cm/year) per 100 mg/day higher ascorbic acid intake in tertiles of the WHR associated SNP score**

*Abbreviations: WHR score, sum of waist-hip ratio associated risk-alleles*

*Estimates were calculated in MONICA, DCH and INTER99 using linear regression and pooled using a fixed effect meta-analysis approach.*

*Basic adjustments: Baseline measure of WC, height, sex, age, smoking status,* *alcohol consumption, physical activity, education, menopausal status for women.*

*Participants per tertile (range of risk alleles): T1 N=1,864 (7-13), T2 n=1,540 (14-15) and T3 n=1,453 (16-22).*
